# Analyzing the influence of withering degree on the dynamic changes in non-volatile metabolites and sensory quality of Longjing green tea by non-targeted metabolomics

**DOI:** 10.3389/fnut.2023.1104926

**Published:** 2023-03-14

**Authors:** Xujiang Shan, Qinyan Yu, Le Chen, Shan Zhang, Jiayi Zhu, Yongwen Jiang, Haibo Yuan, Qinghua Zhou, Ji Li, Yujie Wang, Yuliang Deng, Jia Li

**Affiliations:** ^1^Key Laboratory of Tea Biology and Resources Utilization, Ministry of Agriculture, Tea Research Institute, Chinese Academy of Agricultural Sciences, Hangzhou, China; ^2^State Key Laboratory of Tea Plant Biology and Utilization, Anhui Agricultural University, Hefei, China; ^3^College of Environment, Zhejiang University of Technology, Hangzhou, China; ^4^School of Landscape Architecture and Horticulture Sciences, Southwest Forestry University, Kunming, China; ^5^Agriculture and Rural Bureau of Chun'an County, Hangzhou, China

**Keywords:** withering, green tea, metabolomics, electronic tongue, taste, soup color

## Abstract

Withering is an important processing stage in green tea, which contributes to the tea flavor quality. The aim of this work was to comprehensively investigate the changes of chemical features and flavor attributes in Longjing green teas produced with five different withering degrees (moisture content of 75.05, 72.53, 70.07, 68.00, and 64.78%, w.b.). Combined with human sensory evaluation, electronic tongue and chromatic differences analysis, an assessment of the relationship between the withering degree and the sensory quality of Longjing tea was obtained. By using a non-targeted metabolomics approach, 69 significantly differential metabolites were screened. As the withering degree increased, most free amino acids and catechin dimers were increased, largely attributed to the hydrolysis of proteins and catechin oxidative polymerization, respectively. The contents of organic acids as well as phenolic acids and derivatives were reduced. Interestingly, flavone *C*-glycosides decreased overall while flavonol *O*-glycosides increased. The correlation analysis revealed that metabolites such as theasinensin F, theasinensin B, theaflavin, theaflavin-3,3′-gallate, theaflavin-3′-gallate, malic acid, succinic acid, quinic acid, theanine glucoside and galloylglucose had a greater influence on the taste and color of tea infusion (|r| > 0.6, *p* < 0.05). Overall, an appropriate withering degree at a moisture content of around 70% is more favorable to enhance the Longjing tea quality. These results may enhance the understanding of green tea flavor chemistry associated with withering and provide a theoretical basis for green tea processing.

## 1. Introduction

As one of the top three beverages in the world, tea is loved by people everywhere (1, 2). According to different manufacturing processes tea can be divided into six different categories: green tea, black tea, oolong tea, yellow tea, dark tea, and white tea (3). The core difference between the six major tea types is the different degrees of fermentation, and green tea belongs to the non-fermented tea (4). Around the world, green tea is the second largest consumption of tea which sales only after black tea, and it is more popular in Asia (5). High quality green tea is often characterized by aromas such as sweet, floral, and chestnut-like odors, and its umami and unique sweetness aftertaste also make it highly appreciated. In addition, more and more studies have shown that green tea has significant antioxidant and anti-cancer health benefits for the human health (6, 7).

Among various green tea products, Longjing tea is a typical pot-fired green tea, which is one of the top ten famous teas in China. The shaping of Longjing is special in that it is performed in a high temperature environment by constant extrusion in the vertical direction. Unique processing contributes to the flavor characteristics of Longjing tea, such as green bloom color, fragrant scent, brisk and mellow taste, flat and smooth appearance.

The outstanding quality of green tea infusion is greatly influenced by the non-volatile components (8), and it is generally believed that amino acids, tea polyphenols and caffeine have the most influence on the taste of tea infusion. Amino acids mainly provide umami sensation to tea infusion (9), of which glutamic acid is the main source of umami (10). High levels of theanine, a unique amino acid in tea leaves, is also widely studied as umami substance (11). Bitterness and astringency are usually present together to evaluate tea infusion because of the high synergistic relationship of them (12). Caffeine and catechins are the main sources of bitterness and astringency, and their contribution are especially pronounced in green tea (10, 12, 13). Flavonol glycosides also impart astringency and bitterness, on the other hand, they exert a greater impact on the soup color of green tea (14, 15). In addition, organic acids are among the substances that make up the taste of tea infusion, contributing to its acidity and fruity flavor (16).

The tea varieties and the manufacturing processes are two of the most prominent factors affecting the variations of non-volatile components in the tea leaves (17), especially the latter in terms of regulating various processing parameters to form desired aroma and taste characteristics of tea products. Usually, the most critical processing stages of green tea include fixation and drying (3). The main purpose of fixation is to sharply inactive the endogenous enzymes by over-heating and meanwhile to promote the transformation of aroma substances. Drying, as the last step, function of dehydration, shaping, and further increasing the quality. And different green teas have different physical shaping methods, such as pressing and rolling. However, in practice, in addition to these stages, spreading prior to fixation has also been considered as a vital prerequisite for green tea manufacturing. That is because if the large number of fresh leaves after plucking were piled up and an appropriate processing was not timely conducted, the tea leaves were prone to excess oxidation, possibly leading to deterioration in tea quality. Therefore, the fresh leaves after plucking should be well spread out before fixation, during the period of which, the tea leaves undergo a slight withering. In short, withering is the process of dehydration in fresh tea leaves. During this process, changes in oxidase and hydrolase activities are key to the alteration of various non-volatile components and the formation of green tea quality characteristics (3). It is commonly believed that the moisture content of fresh leaves can be used as an indication of the degree of withering.

Despite the crucial role of withering in the formation of overall quality of green tea, there are apparently less attentions focusing on green tea withering compared to other stages in green processing such as fixation and drying (18, 19), or the withering stage of black tea (20, 21). To date, only a few studies have been reported in this context. It has been shown that withering of green tea has a positive effect on quality enhancement, especially to produce famous green tea (22). The effects of different withering temperatures, withering times and light on the changes of amino acids, catechins, caffeine, and gallic acid in green tea have been explained (1, 2). However, these current studies only focused on the changes of some major substances under different withering conditions, which is apparently not complete given the complex phytochemicals of tea leaves. Moreover, the effect of different withering conditions on the overall quality of tea leaves has not been comprehensively elucidated.

Non-targeted metabolomics, as a state-of-the-art tool, enables identification and quantitation of a large number of endogenous metabolites simultaneously, which provides an “unbiased” view of the global metabolome, and therefore has been extensively used in tea research (23–25). In this study, to obtain an in-depth understanding of the influence of withering degree on the quality of Longjing tea, a reasonable assessment was conducted through human bioresponse evaluation combined with electronic tongue characterization and chromatic differences analysis. Further, non-targeted metabolomics was also used to comprehensively describe the chemical distinction of non-volatile metabolites in finished Longjing teas treated with different withering degrees (withering degrees at moisture of 75.05, 72.53, 70.07, 68.00, and 64.78%, respectively).

## 2. Materials and methods

### 2.1. Chemicals and reagents

Indantrione hydrate, stannous chloride and anthrone were purchased from Macklin biotechnology (Shanghai, China). Chromatography-grade methanol and acetonitrile were purchased from Merck (Darmstadt, Germany). Formic acids (≥96%), acetic acid (≥96%) were purchased from Sigma-Aldrich (St. Louis, MO, United States). Authentic standards were purchased from Sigma-Aldrich (St Louis, MO, United States), Yuanye bio-technology (Shanghai, China), Jinsui biotechnology (Shanghai, China), J&K scientific (Beijing, China) and ZZBIO (Shanghai, China). Diagnostic solution for electronic tongue correction was purchased from Evensen Technology Co., Ltd. (Tianjin, China).

### 2.2. Tea processing

Fresh tea leaves (one bud with one leave) of cultivar *C. sinensis* cv. Jiukengzao were used as raw materials for Longjing tea processing. The tea leaves were harvested in Chun’an, Zhejiang Province, China. Firstly, a total of 37.5 kg of fresh leaves were divided into five equal portions and spread in a thickness of 2–3 cm. The overall environment had a temperature of 19–22°C and a relative air humidity of 55%. Each portion were withered until different moisture contents of leaves were reached, i.e., 75.05, 72.53, 70.07, 68.00, and 64.78% (w.b.), marked as W1, W2, W3, W4, and W5. The withering time used was about 0.5, 5.0, 7.0, 9.0, and 11.5 h, respectively. The withering process was terminated by immediate fixation after reaching the desired moisture content. Then tea leaves of five withering degrees were subjected to subsequent processing including fixation and drying following same parameters. The fixation was performed using an automatic flat tea stir-frying machine (6CCB-981ZD, Zhejiang Yinqiu Machinery Co., Ltd) at a temperature of 205°C. The final drying process was carried out using a pot-drying machine (6CMG-72, Zhejiang Yinqiu Machinery Co., Ltd) at 180°C to reduce the moisture of the samples to 6–7%. The obtained Longjing tea samples were stored in a-20°C freezer before analysis.

### 2.3. Sensory evaluation

The sensory quality of tea samples was evaluated by a team of five professional panelists according to the National Standard of China (GB/T23776-2018). Each green tea sample (3.0 g) was brewed in boiling water (150 mL) for 4 min to obtain the infusion used for taste evaluation. The attributes to be examined included appearance, soup color, aroma, taste, and infused leaves. The sensory evaluation was based on a 100-point system, with the total score calculated as 25, 10, 25, 30, and 10% for appearance, soup color, aroma, taste, and infused leaves, respectively, according to GB/T23776-2018. Informed consent of all participants was obtained before the sensory evaluation.

### 2.4. Electronic tongue analysis

An objective taste evaluation of tea infusions was executed according to electronic tongue analysis. The whole electronic tongue system was composed of one Ag/AgCl reference electrode and seven chemical sensors, which were AHS (sourness), NMS (umami), CTS (saltiness), ANS (sweetness), SCS (bitterness), PKS, and CPS (comprehensive taste). Two infusions were prepared for each tea sample, and each infusion was measured for three times to minimize errors.

### 2.5. Chromatic differences analysis of tea liquors

The color quality of tea liquors was evaluated by chromatic differences analysis using a colorimeter spectrophotometer (CM-5, Konica Minolta (China) Investment Co., Ltd.), through the measurement of L* (brightness), a* (+a*, red, −a*, green), b* (+b*, yellow, −b*, blue), and C* (color saturation). Each sample of tea liquors was measured three times to ensure its accuracy.

### 2.6. Analysis of major tea chemical constituents

Gallic acid, caffeine and catechins were detected as previously descried (26). Briefly, they were separated by using solvent A (2% acetic acid in water) and solvent B (100% acetonitrile) on a symmetry C18 column (4.6 × 250 mm, 5 μm, Waters) at column of 35°C, and were detected on a high performance liquid chromatography (HPLC) system (Waters 2,469, United States) equipped with an ultraviolet detector, with the detection wavelength set at 280 nm. The content of total amino acids and total soluble sugars was determined by National Standard of China (GB/T 8314-2013) and the anthrone–sulfuric method (27), respectively.

### 2.7. Non-targeted metabolomics analysis

The non-targeted metabolomics analysis was referenced from our previous studies (23–25). The tea samples were first ground into tea powder using a powdering machine, then extracted by 70% methanol, and each tea sample was extracted three times in parallel. Mixing equal amount from each tea sample to obtain quality control (QC) samples. Metabolomics data was collected by separating metabolites using a Dionex Ultimate 3000 ultra-high performance liquid chromatography (UHPLC) system (Thermo Fisher, CA, United States) equipped with an ACQUITY UPLC HSS T3 column (2.1 × 100 mm, 1.8 μm, Waters, MA, United States) at 35°C and mass spectrometric acquisition using an electrospray ionization-quadruple-Orbitrap mass spectrometry system (Q-Exactive, Thermo Fisher, CA, United States). 0.1% formic acid aqueous solution was used as mobile phase A, and 0.1% formic acid in acetonitrile as mobile phase B. High resolution (FWHM = 70,000) full scan data was acquired in ESI (−) mode with mass-to-charge ratio (*m/z*) range of 100–1,000. The spray voltage was 3.5 kV for ESI (−). The capillary temperature, probe heater, sheath gas and auxiliary gas were 320°C, 320°C, 45 arb, and 10 arb, respectively. MS/MS fragments were acquired at a collisional energy of 40 eV. QC samples were analyzed every 10 samples throughout the liquid chromatography-mass spectrometry (LC–MS) running sequence. The metabolite annotation was performed according to several principles, including queries against database of HMDB,^1^ PubChem^2^ and published literature, exact mass measurement (mass error < 5 ppm), MS/MS fragments, LC retentions, and validation by authentic standards.

### 2.8. Data analysis and statistics

The obtained raw LC–MS data were aligned, and feature extracted using XCMS 3.4.1 software to generate *m/z*, retention time (RT) and peak intensity of all detected ions. The obtained data were subsequently normalized, and this normalized data indicated the relative abundance of metabolites. Missing values were processed according to the 80% principle, and QC samples were evaluated as previously described (28). Metabolites ions with RSDs less than 20% in QC samples were retained for subsequent analysis. SIMCA-P 14.1 (Umetrics AB, Umeå, Sweden) was used to perform principal component analysis (PCA) and partial least square-discriminate analysis (PLS-DA). Statistical significance was evaluated by one-way ANOVA with LSD *post hoc* test, using SPSS 23.0 (IBM, New York, United States). Visualization of heat maps and hierarchical clustering analysis was performed using Toolbox fox biologists (version 1.09). The algorithm PearsonDist was used for correlation analysis. The taste radar plot and correlation heatmap were generated through using the website-based tool chiplot.^3^

## 3. Results and discussion

### 3.1. Comparison of sensory quality of Longjing teas treated with different withering degrees

Supplementary Table S1 showed the results of sensory evaluation of Longjing green teas with different withering degrees including an assessment of appearance, liquor color, aroma, taste, and infused leaves. As the withering degree increased, the Longjing teas quality showed a trend of increasing and then decreasing, and a relatively higher total quality score was obtained for tea treated with W3. A similar trend was also observed for taste and aroma scores. In terms of soup color, as the withering degree increased the soup color turned greener.

### 3.2. Electronic tongue and chromatic differences analysis

**Figure 1:**
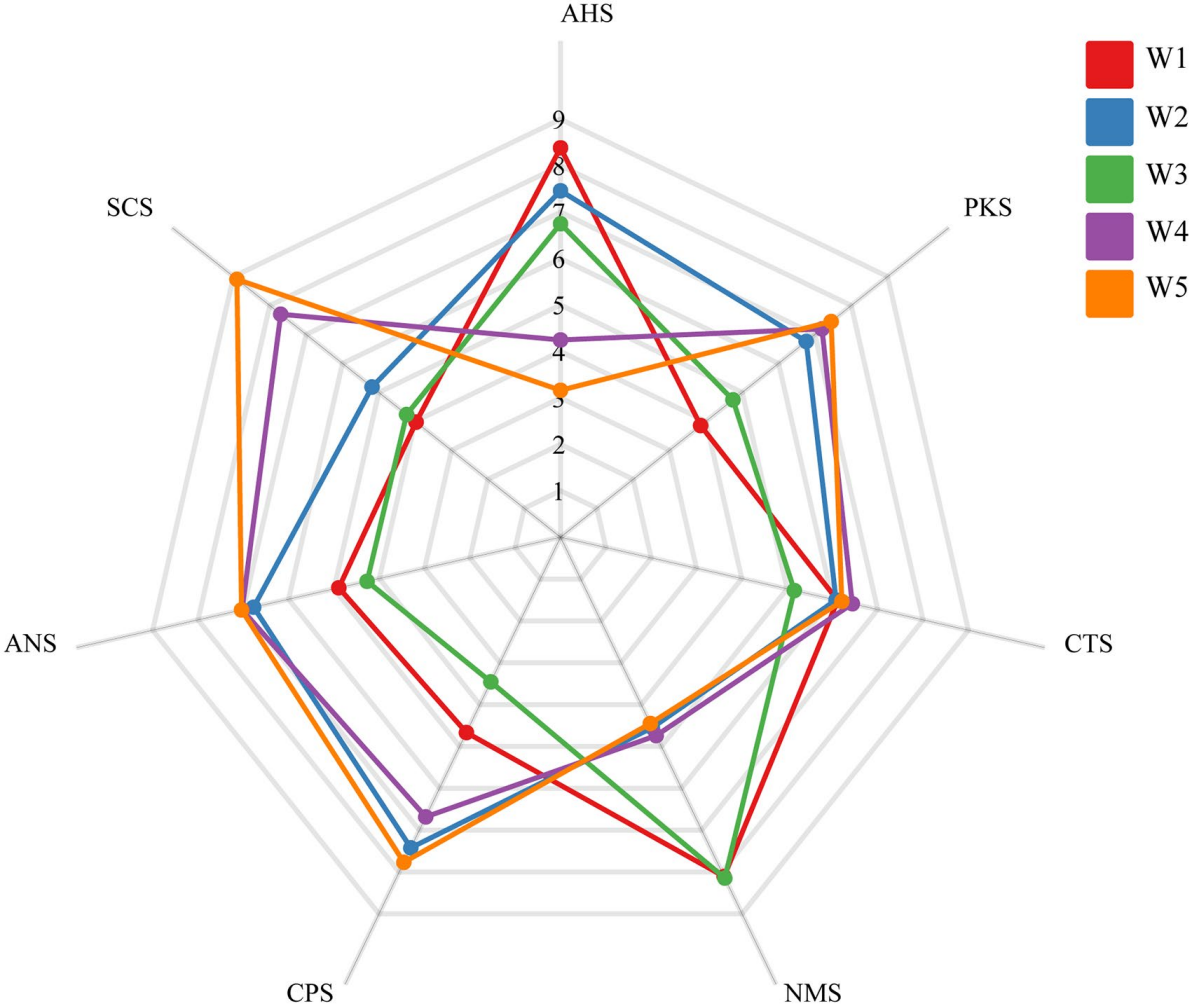
Radar charts of the taste profiles of Longjing tea infusions with different withering degrees, presented using responses of electronic tongue sensors. Tea samples with different withering degrees (the actual measured moisture contents at 75.05, 72.53, 70.07, 68.00, and 64.78%) were expressed as W1, W2, W3, W4, and W5.

**Figure 2:**
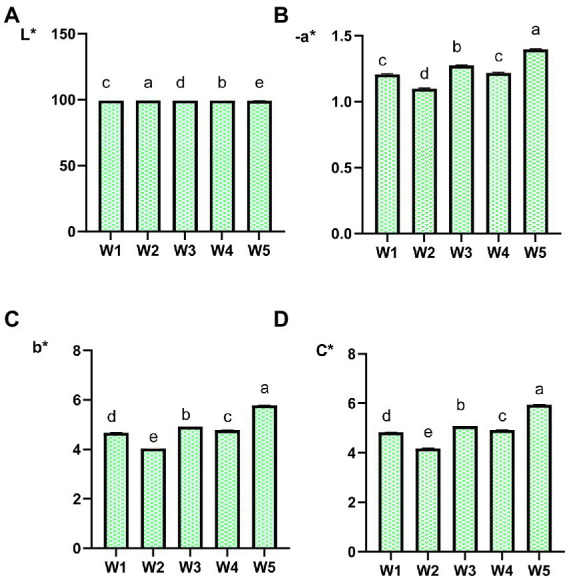
Distinction in color attributes L*, −a*, b*, and C* between Longjing tea infusions with different withering degrees. Different letters indicate a significant difference. And significant differences were obtained by one-way ANOVA with LSD *post hoc* test. *p* < 0.05 is considered as significant. A, L* (brightness). B, −a* (green). C, b* (yellow). D, C* (color saturation).

To evaluate the taste and color attributes of tea infusions in a more objective pattern, electronic tongue responses and chromatic differences were measured. The taste profiles and soup color scores of Longjing teas with different withering degrees were shown in Figures 1, 2.

The taste radar plot showed that the acidity response (AHS) of green tea gradually decreased with the increase of withering degree (*p* < 0.001). The highest acidity was assigned to tea sample W1 and reached 8.38, while the lowest acidity was only 3.12 for W5. Overall, the acidity score of the tea samples treated with relatively heavy withering (W4 and W5) were significantly lower than other three treatments. But acidity was not reflected differently in the sensory evaluation, probably due to much less taste intensity of acidity than that of bitterness and umami. In terms of bitterness response (SCS), the highest score was as high as 8.90 for the tea sample W5 with the strongest withering degree, followed by W4. The bitterness values of the tea samples with light and medium withering (W1, W2, and W3) were significantly lower than those of W4 and W5 (*p* < 0.001) but were not significantly different from each other. It suggested that higher content of taste-active compounds related with bitterness in green tea may be produced in the heavily withered tea samples. The contribution of sweetness response (ANS) to the tea infusion was less pronounced. There was no significant difference in ANS intensities among five treatments. Significantly higher umami response (UMS) scores were assigned to green teas treated with relatively light and medium withering degree (W1 and W3) which both exceeded 8 (*p* < 0.01).

On the other hand, the chromatic values (−a*, b*, and C*) of green tea infusions showed an overall increasing trend as the withering degrees increased. This also implied that increased withering would make the infusion color become more yellowish green, which was consistent to the result of sensory evaluation.

### 3.3. Contents of main constituents of Longjing tea with different withering degrees

The contents of gallic acid, caffeine, eight catechin monomers, total soluble sugars, and total free amino acids, obtained by quantitative analysis, are demonstrated in Table 1. Epigallocatechin gallate (EGCG) was found to be the dominating component in Longjing tea (7.93–8.71%), followed by caffeine (CAF) (3.05–3.37%) and epicatechin gallate (ECG) (2.43–2.74%). From W1 to W5, CAF, epicatechin (EC), EGCG, ECG showed a trend of firstly increasing and then decreasing, and there was an overall decrease trend in gallocatechin (GC), epigallocatechin (EGC), gallocatechin gallate (GCG). These changes was likely to be the result of reversible epimerisation between catechins and oxidation catalyzed by polyphenol oxidase (1). Among gallic acid (GA), CAF and the eight catechins, significant difference (*p* < 0.05) was observed only for GC and GCG. Moreover, the content of galloyl catechins was more than 3 times higher than that of degalloyl catechins. The ratio of galloyl catechins to degalloyl catechins was increased overall and there was a significant difference between W4 and the other samples (*p* < 0.05), implying lower degalloyl degradation of galloyl catechins in relative heavily withered tea. The ratio of *cis-*catechins (epicatechins) to *trans*-catechin was significantly lower in W1 (*p* < 0.05), suggesting that greater epimerization conversion may occur in lightly withered tea. Both total free sugars and total free amino acids demonstrated an increasing trend in tea samples with higher withering degree, but only the amount of free amino acids was statistically significant (*p* < 0.01). This result revealed not much evident differences in common main constituents, thus a further investigation covering a broader range of nonvolatile substances is needed to probe the biochemical changes in Longjing teas treated with different withering degrees.

**Table 1:** Contents of major chemical constituents in Longjing tea samples with different withering degrees, including gallic acid, caffeine, catechins, total amino acids and total soluble sugars.

Compounds	W1	W2	W3	W4	W5
Gallic acid (GA) (%)	0.18 ± 0.02	0.17 ± 0.04	0.18 ± 0.01	0.18 ± 0.03	0.17 ± 0.02
Caffeine (CAF) (%)	3.05 ± 0.11	3.13 ± 0.10	3.37 ± 0.15	3.32 ± 0.21	3.27 ± 0.23
Gallocatechin (GC) (%)	0.36 ± 0.04^a^	0.24 ± 0.02^b^	0.23 ± 0.04^b^	0.21 ± 0.01^b^	0.19 ± 0.02^b^
Epigallocatechin (EGC) (%)	1.52 ± 0.25	1.51 ± 0.25	1.5 ± 0.32	1.18 ± 0.08	1.39 ± 0.22
Catechin (C) (%)	0.24 ± 0.010	0.23 ± 0.004	0.24 ± 0.015	0.23 ± 0.012	0.24 ± 0.014
Epicatechin (EC) (%)	1.22 ± 0.07	1.35 ± 0.04	1.4 ± 0.09	1.33 ± 0.11	1.3 ± 0.31
Epigallocatechin gallate (EGCG) (%)	8.08 ± 0.31	8.52 ± 0.27	8.71 ± 0.50	8.52 ± 0.55	7.93 ± 0.76
Gallocatechin gallate (GCG) (%)	0.35 ± 0.01^a^	0.31 ± 0.01^b^	0.32 ± 0.01^b^	0.33 ± 0.01^ab^	0.31 ± 0.01^b^
Epicatechin gallate (ECG) (%)	2.43 ± 0.144	2.65 ± 0.120	2.74 ± 0.09	2.72 ± 0.197	2.57 ± 0.202
Catechin gallate (CG) (%)	0.02 ± 0.0003	0.02 ± 0.0006	0.02 ± 0.0005	0.02 ± 0.0006	0.02 ± 0.0020
Degalloyl catechins (%)	3.33 ± 0.34	3.34 ± 0.27	3.37 ± 0.25	2.96 ± 0.2	3.12 ± 0.16
Galloyl catechins (%)	10.87 ± 0.45	11.5 ± 0.38	11.79 ± 0.59	11.59 ± 0.73	10.83 ± 0.97
Galloyl catechins/Degalloy catechins	3.27 ± 0.2^b^	3.46 ± 0.28^b^	3.51 ± 0.29^b^	3.92 ± 0.1^a^	3.46 ± 0.16^b^
*cis*-catechins/*trans*-catechins	13.77 ± 0.51^b^	17.53 ± 0.92^a^	17.8 ± 1.29^a^	17.27 ± 1.43^a^	17.29 ± 2.18^a^
Total soluble sugars (%) Total free amino acids (%)	5.86 ± 0.22 3.19 ± 0.09^d^	6.20 ± 0.18 3.34 ± 0.03^c^	6.15 ± 0.56 3.72 ± 0.06^a^	6.25 ± 0.53 3.43 ± 0.01^c^	6.16 ± 0.76 3.62 ± 0.03^b^

Data was presented as mean ± SD (*n* = 3). The actual measured moisture contents of different withering degrees were 75.05, 72.53, 70.07, 68.00, and 64.78%, marked as W1, W2, W3, W4, and W5. Different letters indicated statistically significant (*p* < 0.05) between two groups.

### 3.4. LC–MS based metabolomics analysis

**Figure 3:**
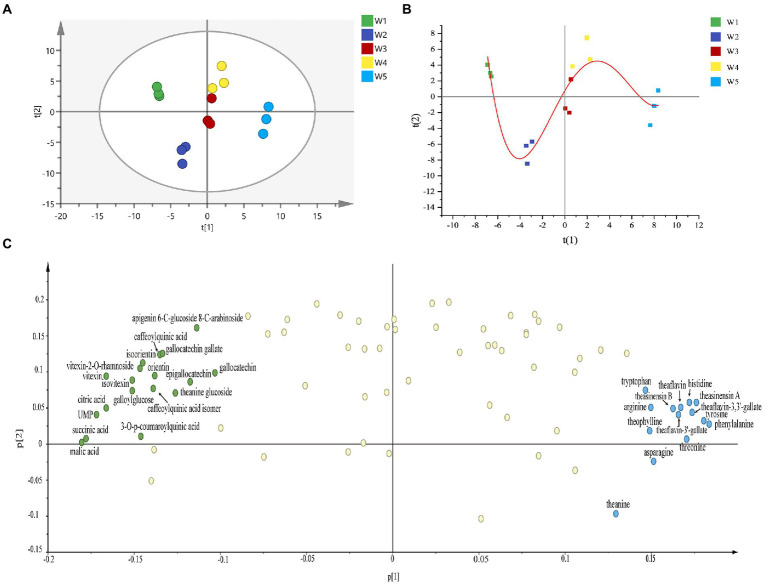
Multivariate statistical analysis of Longjing tea samples with different withering degrees. **(A)** The score plot of principle component analysis (PCA). **(B)** The metabolic trajectory plot. **(C)** The loading plot of PCA model.

In order to comprehensively characterize the metabolic feature in Longjing tea with different withering levels and to identify the key differential metabolites, non-targeted metabolomics analysis based on LC–MS coupled with uni−/multi-variate statistical analysis was applied. The gradient elution method was used to achieve an efficient separation of non-volatile substances from tea leaves within 15 min, as shown in the TIC (Supplementary Figure S1). In addition, the *R*^2^ = 0.99 between two parallel extractions of the same tea sample also indicated the high analytical precision of presented metabolomics analysis (Supplementary Figure S2). All identified metabolites were used for multivariate statistical analysis in this study. As shown in Figure 3A, the unsupervised multivariate analysis PCA model score plot (R^2^X = 0.84) revealed a clear clustering and differentiation of green teas with different withering degrees. The first two major components accounted for 38.3% (PC1) and 30.2% (PC2) of total variance, respectively. The largest distance between W1 and W5 samples was observed, indicating the largest chemical difference between them. On the other hand, W2, W3, and W4 were close in their metabolic profiles. The relative larger differences between the adjacent W1 and W2, W4 and W5 suggested that metabolites may be prone to fluctuate in Longjing tea with light withering and heavy withering treatments. The metabolic trajectory plot (Figure 3B) revealed that Longjing tea samples with different withering degrees showed a wave-like changing trend. The PLS-DA model demonstrated its reliability (R^2^X = 0.901, R^2^Y = 0.983, and *Q*^2^ = 0.934), which used permutation testing with 200 iterations (*R*^2^ = 0.399, *Q*^2^ = −0.609) (Supplementary Figure S3). Furthermore, the PCA loading plot was applied. The PCA loading plot and PCA model score plot are illustrated complementarily to each other. Simply, samples located in a direction of score plot are influenced by the positive correlation of variables lying in the same direction in the loading plot. As demonstrated in Figure 3C, organic acids, catechins, phenolic acids and their derivatives, flavone glycosides, theanine glucoside had a high positive correlation with W1, while on the other hand catechin dimers and amino acids showed great contribution to W5.

**Figure 4:**
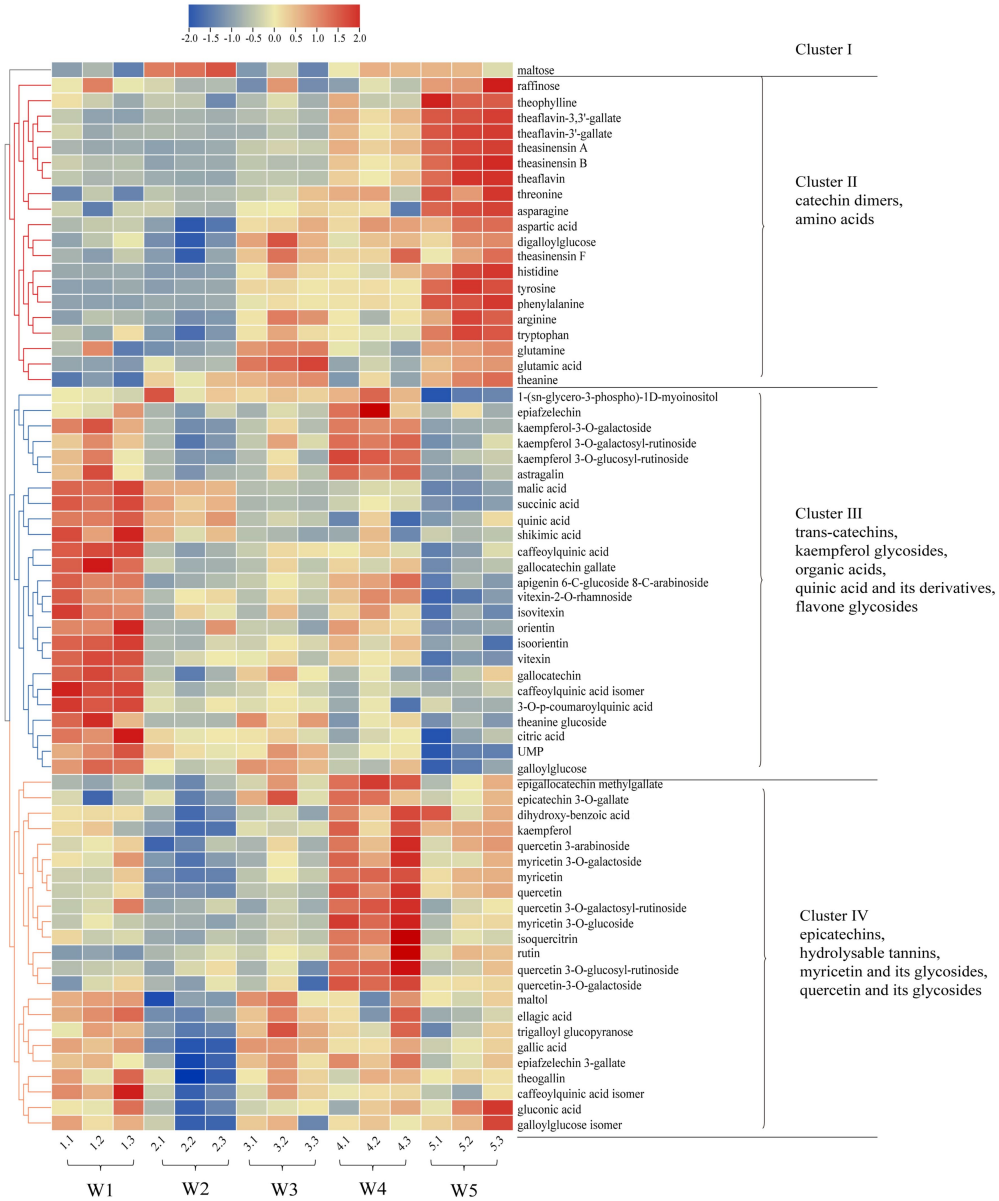
Heatmap of the 69 differential compounds in Longjing tea samples with different withering degrees. Red and blue colors indicate metabolite levels above and below the average of all samples at different withering degrees, respectively. Data are shown as the mean of the relative intensities of three replicates after unit variance (uv) scaling. Hierarchical clustering was performed based on Pearson correlation.

Next, according to the criteria of *p* < 0.05, a total of 69 differential metabolites were screened including 12 amino acids and derivatives, 6 catechins, 6 catechin dimers, 14 phenolic acids, and their derivatives, 21 flavonol and flavonol/flavone glycosides, 3 organic acids and 7 other compounds. Among them, 34 metabolites showed the variable importance in projection (VIP) values >1. The *m/z*, RT, and VIP, MS/MS were reflected in Table 2. Sixty-nine differential metabolites were displayed in heat map (Figure 4), which can more clearly reflect their dynamic changes among Longjing tea samples of five different withering treatments, where the red indicated higher levels whereas the blue indicated lower levels. These differential metabolites were divided and clustered into four groups according to their content distribution in tea samples with five withering treatments, based on hierarchical clustering analysis. First, the metabolite maltose was divided into a separate group. Cluster 2 mainly included catechin dimers and amino acids. The levels of these substances tended to increase, with most metabolites reaching their maximum at the W5. Conversely, there was an overall decrease in differential metabolites in Cluster 3, mainly including kaempferol glycosides, organic acids, flavone glycosides, *trans*-catechins (GCG, GC), quinic acid and its derivatives, etc. Besides, metabolites of Cluster 4 were myricetin, quercetin and their glycosides, epicatechins and hydrolysable tannins, etc. They exhibited a fluctuating trend of firstly falling and then rising. In addition, the color variation in the heat map also showed the greatest metabolic variation in relatively lightly withered teas (W1-W2) and heavily withered teas (W4-W5), which was consistent with the PCA score plot.

**Table 2:** The detailed information of 69 differential metabolites among Longjing green tea samples treated by different withering degrees, including *m/z*, Rt, MS/MS fragments, mass error, *p* value, VIP value of PLS-DA models, and folds change (W5 vs. W1).

No.	Compound	Detected *m/z*	Rt/min	MS/MS	mass error (ppm)	*p* value	VIP	folds change
Amino acids and derivatives								
1	Histidine^a^	154.0612	0.64	93, 137	−2.60	<0.01	0.90	1.88
2	Arginine^b^	173.1033	0.64	131	−2.89	<0.01	1.01	1.34
3	Threonine^a^	118.0497	0.70	72, 74	−1.69	<0.01	0.82	1.46
4	Glutamine^b^	145.0608	0.70	109, 127	0.69	<0.01	1.12	1.14
5	Asparagine^a^	131.0449	0.70	95, 114, 88	−1.53	<0.01	0.91	2.58
6	Glutamic acid^a^	146.0447	0.72	102, 128	−4.10	<0.01	1.40	1.14
7	Aspartic acid^a^	132.0290	0.72	88, 115	−4.50	<0.01	0.92	1.18
8	Theanine^a^	173.0922	0.88	128, 155	−2.30	<0.01	1.05	1.16
9	Theanine glucoside^b^	335.1462	0.90	155, 173, 245	0.90	<0.01	1.01	0.54
10	Tyrosine^a^	180.0657	1.32	72, 93, 119, 163	−1.67	<0.01	0.90	2.09
11	Phenylalanine^a^	164.0705	2.43	97, 137, 147	−3.66	<0.01	0.90	3.34
12	Tryptophan^a^	203.0819	4.80	116, 142, 159	−0.49	<0.01	0.97	1.26
Catechins								
13	Gallocatechin^a^	305.0669	3.89	125, 137, 165, 179, 219, 221, 261, 287	1.40	<0.01	1.03	0.93
14	Epiafzelechin^a^	273.0770	8.14	187, 189, 229, 255	−1.00	<0.05	1.02	0.96
15	Epigallocatechin methylgallate^b^	471.0932	8.51	125, 161, 183, 305	0.10	<0.01	1.07	1.05
16	Epiafzelechin 3-gallate^b^	425.0875	10.80	169, 255, 273	−0.60	<0.01	0.97	0.97
17	Epicatechin 3-*O*-gallate^a^	441.0836	9.27	169, 289, 331, 271, 193, 397	2.90	<0.05	1.18	1.03
18	Gallocatechin gallate^a^	457.0783	7.53	169, 193, 287, 305, 331	3.50	<0.01	0.98	0.81
Catechin dimers								
19	Theaflavin^a^	563.1196	12.20	379, 407, 425, 501, 519, 545, 241, 167, 215	0.20	<0.01	1.08	2.15
20	Theaflavin-3,3′-gallate^a^	867.1432	12.25	125, 169, 241	2.77	<0.01	1.03	2.35
21	Theaflavin-3′-gallate^a^	715.1307	12.26	125, 169, 241, 407	2.30	<0.01	1.13	2.25
22	Theasinensin B^b^	761.1372	4.95	423, 483, 575, 593, 609, 635, 743	1.80	<0.01	1.12	1.94
23	Theasinensin A^b^	913.1485	6.25	285, 423, 573, 591, 743, 761	1.75	<0.01	0.97	2.55
24	Theasinensin F^b^	897.1534	7.76	407, 727, 745	1.60	<0.01	1.03	1.08
Phenolic acids and derivatives								
25	Quinic acid^a^	191.0552	0.78	85, 93, 127, 173	−4.50	<0.01	1.03	0.87
26	Galloylglucose^b^	331.0677	1.48	125, 169, 211, 271	2.20	<0.01	1.05	0.78
27	Galloylglucose isomer^b^	331.0670	1.64	125, 169, 211, 271	1.20	<0.05	0.95	1.02
28	Gallic acid^a^	169.0133	1.92	125	1.30	<0.01	1.15	0.94
29	Caffeoylquinic acid^b^	353.0875	4.80	135,173,179,191	2.10	<0.01	0.94	0.79
30	Digalloylglucose^b^	483.0784	5.50	125, 169, 211,271, 313, 331	0.83	<0.01	1.13	1.09
31	Dihydroxy-benzoic acid^b^	153.0181	6.70	109	−0.65	<0.01	0.97	1.05
32	3-*O-p*-Coumaroylquinic acid^b^	337.0929	7.02	173	0.10	<0.01	1.07	0.84
33	Theogallin^a^	343.0666	1.95	191	−1.60	<0.05	0.88	0.98
34	Caffeoylquinic acid isomer^b^	353.0881	5.80	135,173,179,191	3.68	<0.01	0.92	0.93
35	Caffeoylquinic acid isomer^b^	353.0876	5.90	135,173,179,191	2.20	<0.01	1.12	0.82
36	Trigalloyl glucopyranose^b^	635.0898	7.58	125, 169, 313, 465, 483	2.40	<0.05	1.13	0.95
37	Shikimic acid^a^	173.0451	0.84	73, 93, 111, 137	−2.31	<0.01	1.15	0.86
38	Ellagic acid^a^	300.9990	9.12	229, 257, 284, 201	3.55	<0.05	0.97	0.89
Flavonol and flavonol/flavone glycosides								
39	Quercetin 3-*O*-galactosyl-rutinoside^b^	771.2003	8.30	301, 343, 609	1.84	<0.01	1.02	0.97
40	Quercetin 3-*O*-glucosyl-rutinoside^b^	771.2003	8.54	301, 343, 609	1.84	<0.01	1.08	1.03
41	Quercetin-3-*O*-galactoside^a^	463.0862	9.20	301, 300, 293	−4.32	<0.01	1.00	1.04
42	Myricetin 3-*O*-galactoside^a^	479.0836	7.98	316, 317, 271	2.20	<0.01	0.93	0.99
43	Myricetin 3-*O*-glucoside^b^	479.0837	8.10	316, 317, 271	2.50	<0.01	1.05	1.03
44	Rutin^a^	609.1461	9.00	301, 343	−0.16	<0.01	1.02	1.31
45	Kaempferol 3-*O*-galactosyl-rutinoside^b^	755.2064	9.03	285	3.20	<0.01	0.93	0.91
46	Isoquercitrin^a^	463.0886	9.40	301, 300	0.86	<0.01	1.01	0.98
47	Kaempferol 3-*O*-glucosyl-rutinoside^b^	755.2064	9.50	285	3.20	<0.01	0.97	0.91
48	Quercetin 3-arabinoside ^b^	433.0780	10.08	300, 271, 301, 255	−4.40	<0.01	0.88	1.03
49	Kaempferol-3-*O*-galactoside^b^	447.0933	10.12	255, 284, 285, 327, 357	−0.10	<0.01	0.93	0.86
50	Astragalin^a^	447.0933	10.54	255, 284, 285, 327, 357	−0.10	<0.01	0.91	0.87
51	Myricetin^a^	317.0301	11.43	113, 137, 151, 179	1.26	<0.01	0.96	1.09
52	Quercetin^a^	301.0360	12.35	107, 121, 151, 179	4.00	<0.01	0.99	1.10
53	Kaempferol^a^	285.0404	12.59	227, 239, 211,	−3.5	<0.01	0.92	1.09
54	Apigenin 6-*C*-glucoside 8-*C*-arabinoside^b^	563.1418	7.83	353, 383, 524, 443, 473, 503, 545	2.00	<0.01	0.92	0.83
55	Isoorientin^b^	447.0937	7.90	327, 357, 401, 429	0.89	<0.01	0.92	0.67
56	Orientin^a^	447.0940	8.15	327, 357, 379, 401	1.57	<0.01	0.91	0.66
57	Vitexin-2-*O*-rhamnoside^a^	577.1579	8.93	413, 293, 457	2.80	<0.01	0.89	0.77
58	Vitexin^a^	431.0988	9.00	283, 311, 341	1.16	<0.01	0.88	0.61
59	Isovitexin^a^	431.0987	9.10	283, 311, 341	0.93	<0.01	0.84	0.62
Organic acids								
60	Malic acid^a^	133.0131	0.87	71, 89, 115	−4.70	<0.01	1.02	0.50
61	Citric acid^a^	191.0190	1.15	85, 111, 173	−3.80	<0.01	0.86	0.79
62	Succinic acid^a^	117.0183	1.41	73, 99	−3.42	<0.01	1.04	0.57
Others								
63	Gluconic acid^b^	195.0502	0.72	129, 159, 177	−4.00	<0.05	0.98	1.05
64	Theophylline^a^	179.0564	4.86	135	−2.79	<0.01	1.11	1.31
65	UMP^b^	323.0287	0.79	173, 211, 279, 305, 79, 193	0.40	<0.01	0.97	0.7
66	1-(sn-glycero-3-phospho)-1D-myoinositol^b^	333.0594	0.71	153, 241	0.60	<0.01	1.27	0.92
67	Raffinose^a^	503.1624	0.75	89, 101, 179, 221	2.38	<0.05	1.26	1.07
68	Maltol^b^	125.0232	1.92	57, 81, 97	−0.50	<0.05	1.03	0.91
69	Maltose^a^	341.1087	0.76	113, 119, 143, 161, 179	−0.40	<0.01	1.22	1.16

^a^Confirmed by standards. ^b^Identified based on exact mass and MS/MS.

### 3.5. Metabolites alterations associated with different withering degrees

**Figure 5:**
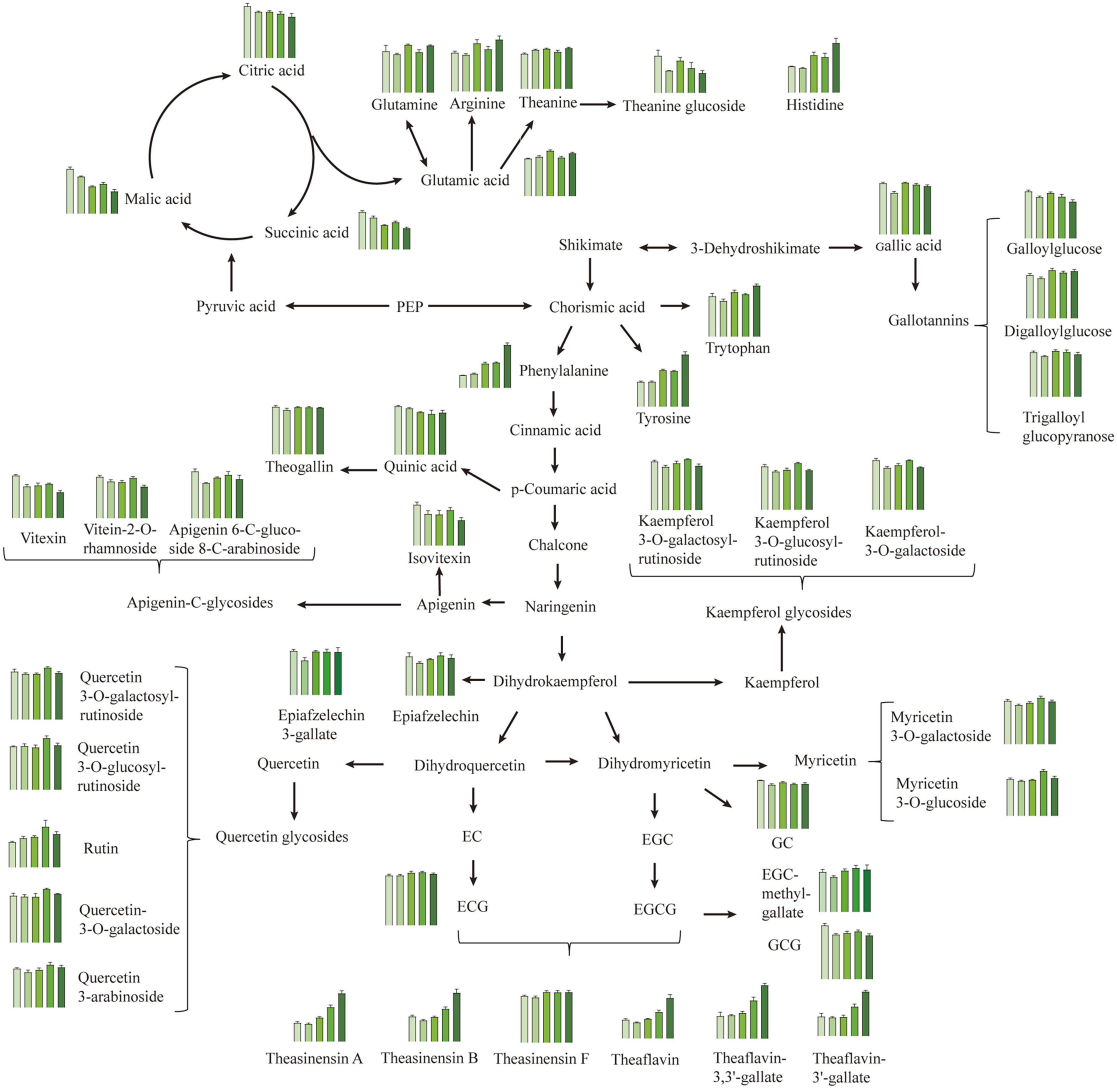
Metabolism pathways of the main metabolites in Longjing tea with different withering degrees, including phenolics, organic acids, and amino acids. The bar plots show the metabolite contents in W1, W2, W3, W4, and W5 from left to right. Data is displayed as mean ± SD using relative abundance which was calculated by normalizing to the total ion intensity. All metabolites shown in the bar plot were significantly differential (one-way ANOVA with LSD *post hoc* test, *p* < 0.05). PEP, phosphoenolpyruvate.

To better assess the metabolic differences in Longjing teas treated with different withering degrees, the metabolic pathway of major metabolites was mapped with the Kyoto Encyclopedia of Genes and Genomes database^4^ which included phenolics, organic acids, amino acids, etc. (Figure 5). All substances in Figure 5 were significantly different (*p* < 0.05), and VIP > 1 for most substances the total amount of each metabolite class was demonstrated in Supplementary Figure S4.

#### 3.5.1. Amino acids

Amino acids are one of the most researched substances in tea taste. Previous research has shown that about 70% of green tea’s umami taste strength is linked to amino acids, especially glutamic acid and theanine (10). In addition, arginine, histidine, phenylalanine and tryptophan contribute to the bitterness (1). In this study, the contents of amino acid compounds showed generally increased pattern in Longjing tea with slight fluctuation as the degree of withering increased (Table 1; Figure 4; Supplementary Figure S4). Hydrolysis of proteins is thought to be responsible for elevated amino acid levels (29). In terms of glutamic acid, its content was generally increased and reached the highest in the W3 with a relative intensity of 3.17 × 10^−4^, followed by the W5. It has been shown that glutamic acid is replenished by protein hydrolysis on one hand, and is depleted as a direct precursor of glutamine, arginine and theanine on the other, which may be the main reason for the fluctuation in its content among five treatments (1). Theanine increased slightly overall, with a trend similar to that of glutamic acid. Although the relative intensity of theanine was high (8.99 × 10^−3^-1.03 × 10^−2^), it may not be a substance that directly brings umami, but rather acts as umami-enhancing compound due to its high taste threshold (11, 12). By far there is still a debate about the changes of theanine during the withering process. Sari, Velioglu et al. revealed that theanine decreases to varying degrees with withering at 32°C (30). However, our results are in agreement with the fluctuating data of Too et al. (2). The result difference between these studies could be due to the effect of different withering conditions, especially the withering temperature. And it was presumed that the higher withering temperature accelerated the hydrolysis of theanine, which may be the main reason for the decrease in theanine content (1). Figure 5 also displayed that the bitter amino acids histidine, phenylalanine, and tryptophan all showed a significant increase at the W5, especially phenylalanine which had a fold change (FC) of 3.34 compared to the W1 (Table 2). Interestingly, by contrast, theanine glucoside, a new theanine derivative, was significantly decreased in Longjing teas with increased withering degree, whose FC was 0.54 (Table 2). And its changing pattern was almost consistent with that of the umami score in electronic tongue response.

#### 3.5.2. Catechins and catechin dimers

Catechins are considered to be the most characteristic metabolites in tea (10, 31), and present a huge role in the bitterness and astringency of the tea infusion (13, 32), especially in green tea. A significant decrease in GC and GCG was observed by metabolomics, which agreed with the result obtained by HPLC analysis (Table 1; Figure 5). Other detected catechin and its derivatives with significant differences such as epicatechin 3-*O*-gallate, epigallocatechin methylgallate, epiafzelechin and epiafzelechin 3-gallate showed a slight increasing trend (*p* < 0.05, Figure 5). However, the overall variation of catechins was not significant (*p* > 0.05, Supplementary Figure S4). On the other hand, theaflavins, which are widely believed to be metabolites unique to black tea, formed by the enzymatic oxidation of catechins during tea fermentation (33), were detected in green tea in the present study. Besides, other catechin dimers including theasinensin A, theasinensin B and theasinensin F were also detected. This result was similar to previous studies, where theaflavins had been found in fresh tea leaves (25, 34). The presence of theaflavins in unfermented green tea was mainly due to the vulnerability of polyphenols to oxidative stress and, to a lesser extent, to the plant’s defense system after plucking (35). In contrast to catechins, despite the relative low contents of these catechin dimers, they showed a significant evident increase in total amount (Supplementary Figure S4). All individual compounds of the catechin dimer had FC values above 1.9, except for theasinensin F (Table 2).The significant increase in catechin dimers content in W5 may be due to the reduction of water caused by long-time withering, which allowed the conversion of low-molecular phenol oxidase to high-molecular phenol oxidase with higher catechol oxidase activity (36, 37). Although the variability of the multiple catechin dimers was significant, the content of catechin dimers was low (The difference between the catechin dimers and catechin monomers was about 2 orders of magnitude in MS intensity).These substances have been proven to be taste-active compounds imparting bitterness and astringency to the tea infusion (38). On the basis of conventional bitter components such as CAF, EGCG without significant changes, we speculated that the significant increase in catechin dimer content may be one of the reasons contributing to the high scores of the electronic tongue bitterness sensor in samples W4 and W5. In addition, previous studies also mentioned that the oxidation of catechins would make the soup yellow, which also verified the rise in b-value at the W5 (39).

#### 3.5.3. Phenolic acid and derivatives

Phenolic acids and derivatives are important antioxidants in tea as well as the main substances for the synthesis of flavonols and catechins (40, 41). The total content of phenolic acids and derivatives showed decreased levels in Longjing tea as the withering degree increased (Supplementary Figure S4). Quinic acid and theogallin were the dominating differential phenolic acids in analyzed Longjing teas, their content percentage in the total differential phenolic acid reached 37.7 and 31.9%, respectively. Quinic acid was significantly decreased in tea samples with medium and heavy withering degrees (W3, W4, and W5). The contents of gallic acid as well as several hydrolysable tannins such as galloylglucose and trigalloyl glucopyranose were slightly higher in tea samples with light and medium withering degrees (W1 and W3) (*p* < 0.05, Figure 5). Usually, these substances are one of the sources of bitterness and astringency in the tea infusion (10). However, there are now some studies that mention their other contributions to taste. Gallic acid and theogallin have been shown to have an enhancing effect on the umami of Japanese matcha (11). Besides, in Han’s study these substances were correlated with the grade of Huangshan Mao Feng green tea and gallotannins were more often found in higher grades of green tea (42). The potential effect of these substances on taste may explain the higher umami scores in Longjing tea with light and medium withering degrees.

#### 3.5.4. Flavonol and flavone/flavonol glycosides

Flavonol and flavone/flavonol glycosides are one of the dominant phenolic components in tea and have been widely studied for their strong antioxidant activity and potential health benefits (43, 44). Their content was lower compared to that of catechins. Herein, except for kaempferol *O*-glycosides, all species of flavonol *O*-glycosides presented a relative content of less than 5 × 10^−4^, and flavone *C*-glycosides such as vitexin and orientin had an even lower content. Although occurred in low abundance, flavone/flavonol glycosides have been identified as key compounds that produce a velvety-like astringency in tea infusion due to their extremely low taste presentation threshold (29, 32) and increase the bitter taste by interacting with caffeine (10). Interestingly, in the present study, it was found that the variations among five withering treatments was associated with linkage pattern of glycosides. The relative content of flavonol *O*-glycosides increased overall as the withering degrees increased, such as rutin, quercetin-3-*O*-galactoside and myricetin 3-*O*-glucoside. There was a fluctuating increase in flavonol *O*-glycosides, and a similar situation was observed in Yue’s study (45). However, flavone *C*-glycosides were dominantly decreased, such as vitexin and isovitexin, as demonstrated in Figure 5 and Supplementary Figure S4. This is different from the results of Yue’s study, and the reason for this may be considered because our test samples were finished teas, which were subsequently also affected by the fixation and drying. Among the flavonol glycosides, rutin showed the maximum variation, with its relative content reaching more than 1.5 times in the heavily withering sample W4 compared to the lightly withering sample W1 (Figure 5). We speculated that the rise in the content of these substances in the heavily withered tea samples (W4 and W5) led to their higher SCS response (bitterness) intensities in the electronic tongue (Figure 1). In addition to functioning as taste-active compounds, flavonol glycosides have showed a strong effect on the green tea color. Quercetin itself shows a slight yellowish green color and is significantly associated with the color of tea infusion (14). Possibly attributed to the increase in the content of flavonol glycosides, the −a* and b* values were increased along with increased withering degrees, resulting in the color differences in Longjing tea infusions (Figure 2).

#### 3.5.5. Organic acid

Organic acids in tea are important intermediate metabolites in its tricarboxylic acid cycle and shikimic acid pathway, which plays an important role in the acidity and the fruitiness of green tea (16, 46). The contents of malic acid, succinic acid and citric acid were significantly different in five withering treatments, decreased for 49.7, 42.9 and 20.5%, respectively, in W5 compared to W1 (Figure 5). This change also contributed to the lower acidity response scores (AHS) as the withering degrees increased (Figure 1). Besides, succinic acid has been reported as umami-enhancing compound (11). Thus, the higher expression of organic acids in the lightly withered tea W1 may have a synergistic effect with umami.

### 3.6. Correlation analysis

**Figure 6:**
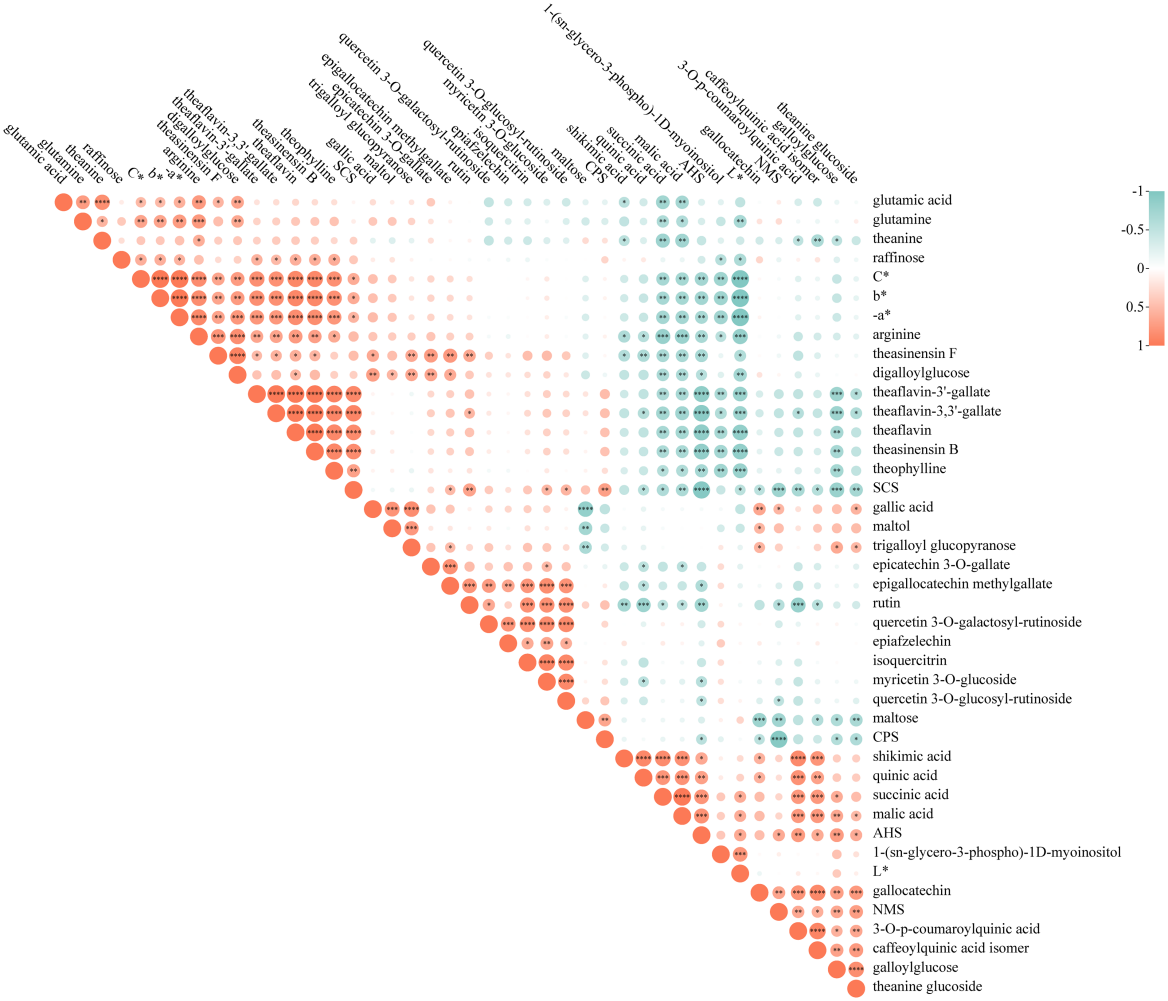
Correlation matrix analysis between key differential metabolites (*p* < 0.05, VIP > 1) and sensory indicators illustrated as heatmap. The correlation coefficient values were ranged from −1 (colored in green) to 1 (colored in red). A larger circle means a stronger correlation and a smaller circle means a weaker correlation. Correlation analysis was performed by PearsonDist. *p < 0.05, **p < 0.01, ***p < 0.001, and ****p < 0.0001.

Figure 6 is a heat map visualizing the correlation between the tea taste, soup color and key metabolites (VIP > 1, *p* < 0.05). The green color represented negative correlation whereas the red represented positive correlation. As can be seen from the figure, the correlation between theasinensin F, theasinensin B, theaflavin, theaflavin-3,3′-gallate and theaflavin-3′-gallate was strong. In particular, the correlations coefficients of theasinensin B with theaflavins were all above 0.97 (*p* < 0.001). The main reason was that these substances are produced by the oxidative polymerization of catechins. Flavonol glycosides showed not only high correlations with each other, but also with catechins, especially with epigallocatechin methylgallate, with a correlation coefficient as high as 0.7 (*p* < 0.01). In addition, multiple classes of substances showed strong correlations with organic acids. For example, organic acids were strongly positively correlated with multiple phenolic acids (*r* > 0.6, *p* < 0.05), and negatively correlated with amino acids and catechin dimers (|r| > 0.6, *p* < 0.05).

All of the color difference indicators showed significant correlations with theasinensin F, theasinensin B and three theaflavins, among which L* showed a negative correlation (|r| > 0.6, *p* < 0.05), − a*, b*, and C* showed positive correlations (*r* > 0.6, *p* < 0.01). This result implied that oxidation products of catechins can affect the infusion color of green tea to some extent. In terms of taste, rutin, theasinensin F and three theaflavins displayed a strong positive correlation with bitterness response (SCS, *r* > 0.6, *p* < 0.01). Rutin has been reported to be bitter and astringent and could enhance the bitterness of caffeine (10). Theasinensin F is a bitter and astringent substance (38). While theaflavin is generally considered to be an astringent substance, and from our results it is possible that theaflavin could exert a synergistic effect on bitterness. The main substances negatively correlated with SCS were malic acid, succinic acid, galloylglucose and theanine glucoside (|r| > 0.6, *p* < 0.05). In addition, most of above-mentioned substances had a strong correlation with acidity response (AHS), but the direction of correlation was opposite. The positive correlation of AHS with malic acid and succinic acid was apparently extra high (*r* > 0.7, *p* < 0.01). Phenolic acids such as quinic acid and coumaroylquinic acid also exhibited a strong positive correlation with AHS (*r* > 0.6, *p* < 0.05). In particular, metabolites theanine glucoside and galloylglucose showed positive correlations with umami response (NMS, *r* > 0.6, *p* < 0.01). It is thus speculated they might be potential factors enhancing umami. However, further experimental data are still needed to confirm this. Because of the complexity of tea infusion, it is important to consider the interactions between tastes. Figure 6 reveled that bitterness showed a strong negative correlation to acidity and umami (|r| > 0.7, *p* < 0.001), which also reflected a certain antagonistic effect between them. On the other hand, umami and acidity seemed to show a synergistic effect (*r* > 0.6, *p* < 0.05). Such potential taste interaction may explain the lower umami score of W5 despite higher content of amino acids. In general, these highly correlated substances such as theasinensin F, theasinensin B, theaflavin, theaflavin-3,3′-gallate, theaflavin-3′-gallate, rutin, malic acid, succinic acid, theanine glucoside and galloylglucose may serve as vital regulatory metabolites related with different withering degrees, which influenced the flavor quality (soup color and taste) of Longjing tea. From the combination of all the results we obtained, medium withering (W3, moisture content of 70.07%, w.b.) was more favorable to improve the quality of tea leaves.

## 4. Conclusion

In this study, a comprehensive and objective analysis of Longjing tea with different withering degrees was conducted by sensory evaluation, electronic tongue and chromatic differences analysis. Subsequently, 69 differential metabolites were screened in finished Longjing teas treated with different withering degrees by non-targeted metabolomics analysis. Metabolic pathways of significantly differential metabolites were mapped. As the withering degree increased, the relative content of most free amino acids increased attributed to the hydrolysis of proteins. The overall change in catechins was not significant, but a significant and well-marked increase in their oxidation polymerization products, including theasinensins and theaflavins, was observed. And the contents of organic acids as well as phenolic acids and derivatives were reduced. Interestingly, flavone *C*-glycosides decreased overall while flavonol *O*-glycosides increased. By correlation analysis, it was found that metabolites including rutin, theasinensin B, theaflavin, theaflavin-3,3′-gallate, theaflavin-3′-gallate, malic acid, succinic acid, quinic acid and coumaroylquinic were the compounds which caused greater effects on the quality of Longjing tea with different withering degrees. In addition, we speculate that theanine glucoside and galloylglucose were likely to function as potential substances affecting umami. In the future, more efforts are needed to confirm the specific effects of these substances on tea taste quality. Collectively, this paper provides a comprehensive description of the quality as well as phytochemical characteristics of five finished Longjing green teas treated with different withering degrees by integrated analysis of various approaches. An appropriate withering degree at about 70% of moisture content was helpful to improve the overall quality of green tea. The result of this study is expected to provide some practical implications in Longjing tea processing.

## Data availability statement

The original contributions presented in the study are included in the article/Supplementary material, further inquiries can be directed to the corresponding authors.

## Author contributions

XS: methodology, investigation, validation, formal analysis, and writing–original draft. QY: investigation, formal analysis, and resources. LC: investigation and resources. SZ: investigation and resources. JZ: formal analysis. YJ: funding acquisition and supervision. HY: supervision and data curation. QZ: methodology. JiL: resources. YW: writing–review and editing. YD: investigation and resources. JiaL: conceptualization, methodology, formal analysis, writing–original draft, and writing–review and editing. All authors contributed to the article and approved the submitted version.

## Funding

This work was supported by grants of the Science and Technology Innovation Project of the Chinese Academy of Agricultural Sciences (No. CAAS-ASTIP-TRICAAS), the Natural Science Foundation of China (No. 31700616, No. 42277275), the Natural Science Foundation of Zhejiang Province (No. LY20B070009), and the Local Scientific and Technological Cooperation Project of Agriculture and Rural Bureau of Chun’an County and Chinese Academy of Agricultural Sciences Tea Research Institute.

## Conflict of interest

The authors declare that the research was conducted in the absence of any commercial or financial relationships that could be construed as a potential conflict of interest.

## Publisher’s note

All claims expressed in this article are solely those of the authors and do not necessarily represent those of their affiliated organizations, or those of the publisher, the editors and the reviewers. Any product that may be evaluated in this article, or claim that may be made by its manufacturer, is not guaranteed or endorsed by the publisher.

## Footnotes

^1^ http://www.hmdb.ca/

^2^ https://pubchem.ncbi.nlm.nih.gov/

^3^ https://www.chiplot.online/

^4^ https://www.Kegg.jp/kegg/pathway.html
