# Supplementary material for: Analyzing the influence of withering degree on the dynamic changes in non-volatile metabolites and sensory quality of Longjing green tea by non-targeted metabolomics
Source: Front Nutr. 2023 Mar 14;10:1104926. doi: 10.3389/fnut.2023.1104926 (PMC10043258; doi:10.3389/fnut.2023.1104926)

Analyzing the influence of withering degree on the dynamic changes in non-volatile metabolites and sensory quality of Longjing green tea by non-targeted metabolomics

Xujiang Shan^a,b‡^, Qinyan Yu^a,‡^, Le Chen^c^, Shan Zhang^a,d^, Jiayi Zhu^a^, Yongwen Jiang^a^, Haibo Yuan^a^, Qinghua Zhou^c^, Ji Li^e^, Yujie Wang^b,^*, Yuliang Deng^a,^*, Jia Li^a,^*

^a^ Key Laboratory of Tea Biology and Resources Utilization, Ministry of Agriculture, Tea Research Institute, Chinese Academy of Agricultural Sciences, Hangzhou 310008, China

^b^ State Key Laboratory of Tea Plant Biology and Utilization, Anhui Agricultural University, Hefei, 230036, China

^c^ College of Environment, Zhejiang University of Technology, Hangzhou 310014, China

^d^ School of Landscape Architecture and Horticulture Sciences, Southwest Forestry University, Kunming 650224, China

^e^ Agriculture and Rural Bureau of Chun'an County 311700, China

* Correspondence should be addressed to:

Dr. Jia Li, Email: [jiali1986@tricaas.com](mailto:jiali1986@tricaas.com);

Yuliang Deng, Email: [donny@tricaas.com](mailto:donny@tricaas.com)

Dr. Yujie Wang, Email: [wangyj@ahau.edu.cn](mailto:wangyj@ahau.edu.cn)

Author contribution:

^‡^ Xujiang Shan and Qinyan Yu contributed equally to this study.

**Table S1** Sensory evaluation of Longjing green tea treated by different withering degrees.

| Withering water content | Appearance (25%) | | Liquor color (10%) | | Aroma (25%) | | Taste (30%) | | Infused leaf (10%) | | Total quality score |
| --- | --- | --- | --- | --- | --- | --- | --- | --- | --- | --- | --- |
|  | comment | score | comment | score | comment | score | comment | score | comment | score |  |
| 75.1%  (W1) | Flatter, smooth, straight, still tender green | 88 | Light soft green, bright | 91 | High, floral fragrance | 91 | Fresh, mellow, and slightly astringent | 91 | Tender, thick, bright green | 91 | 90.25 |
| 72.5%  (W2) | Flatter, smoother, straighter, still tender green | 89 | Light soft green, clear and bright | 92 | High, floral fragrance | 92 | Fresh and mellow | 92 | Tender, thick, bright green | 91 | 91.15 |
| 70.1%  (W3) | Flatter, smoother, straighter, more tender green | 91 | Younger green, clearer and brighter | 90 | Highly fresh, floral fragrance | 93 | Fresh and mellow | 93 | Tender, thick, bright green | 91 | 92.00 |
| 68.0%  (W4) | Flatter, smoother, straighter, more tender green | 90 | Light soft green, bright | 91 | Highly fresh, with floral aroma | 92 | Fresh and mellow | 92 | Tender, thick, bright green | 91 | 91.30 |
| 64.8%  (W5) | Flatter, smoother, straighter, more tender green | 90 | Younger green, clearer and brighter | 89 | Tall, slightly grassy | 90 | Fresh, mellow, and slightly astringent | 91 | Tender, thick, bright green | 91 | 90.30 |

Figure S1 Total ion chromatography (TIC) of Longjing green tea extract acquired using LC-MS in ESI negative mode.


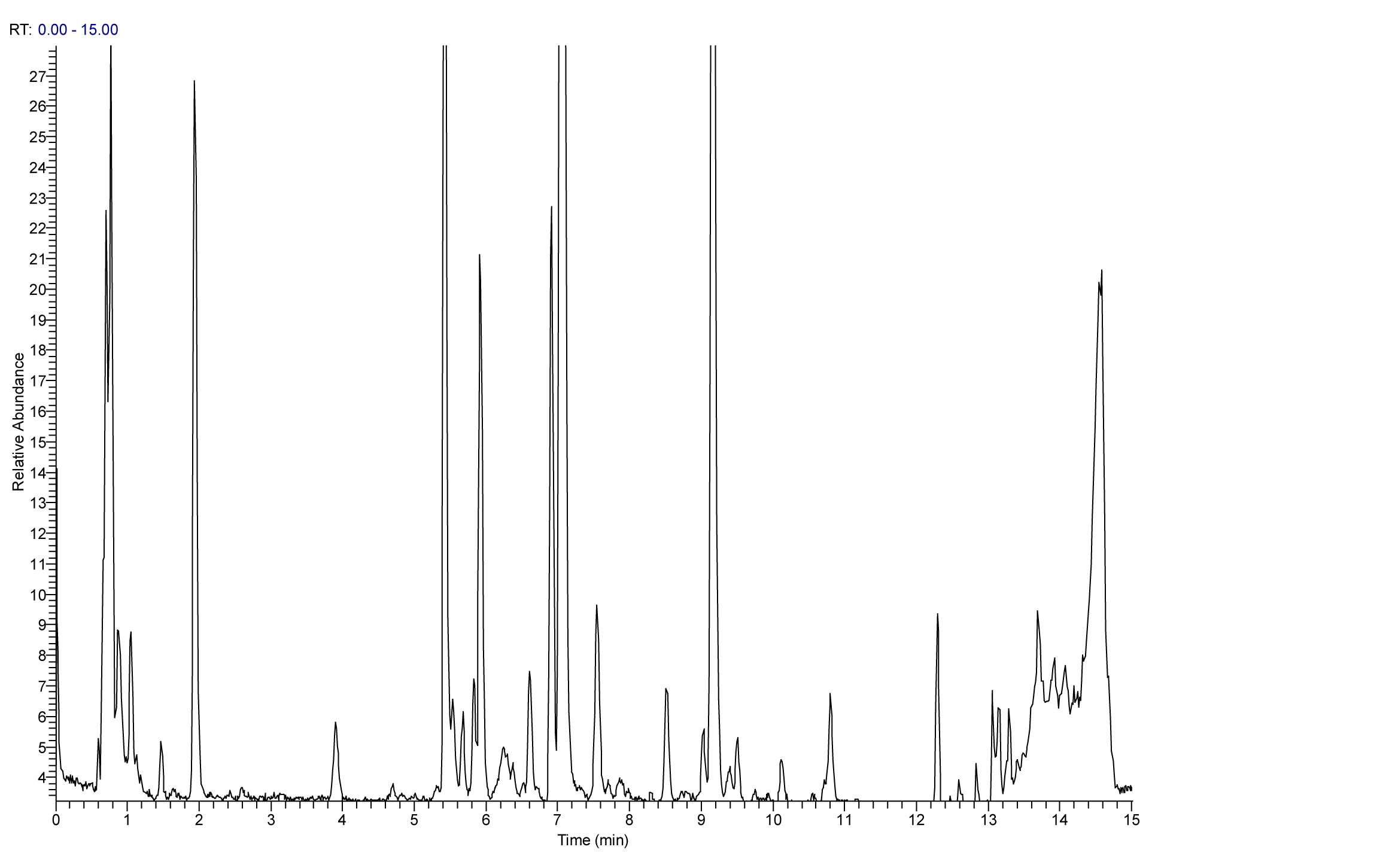


Figure S2 Reproducibility evaluation of metabolomics analysis. Scatter plot of normalized intensities of all detected ions in two parallel extractions. Logarithmic scaling is depicted.


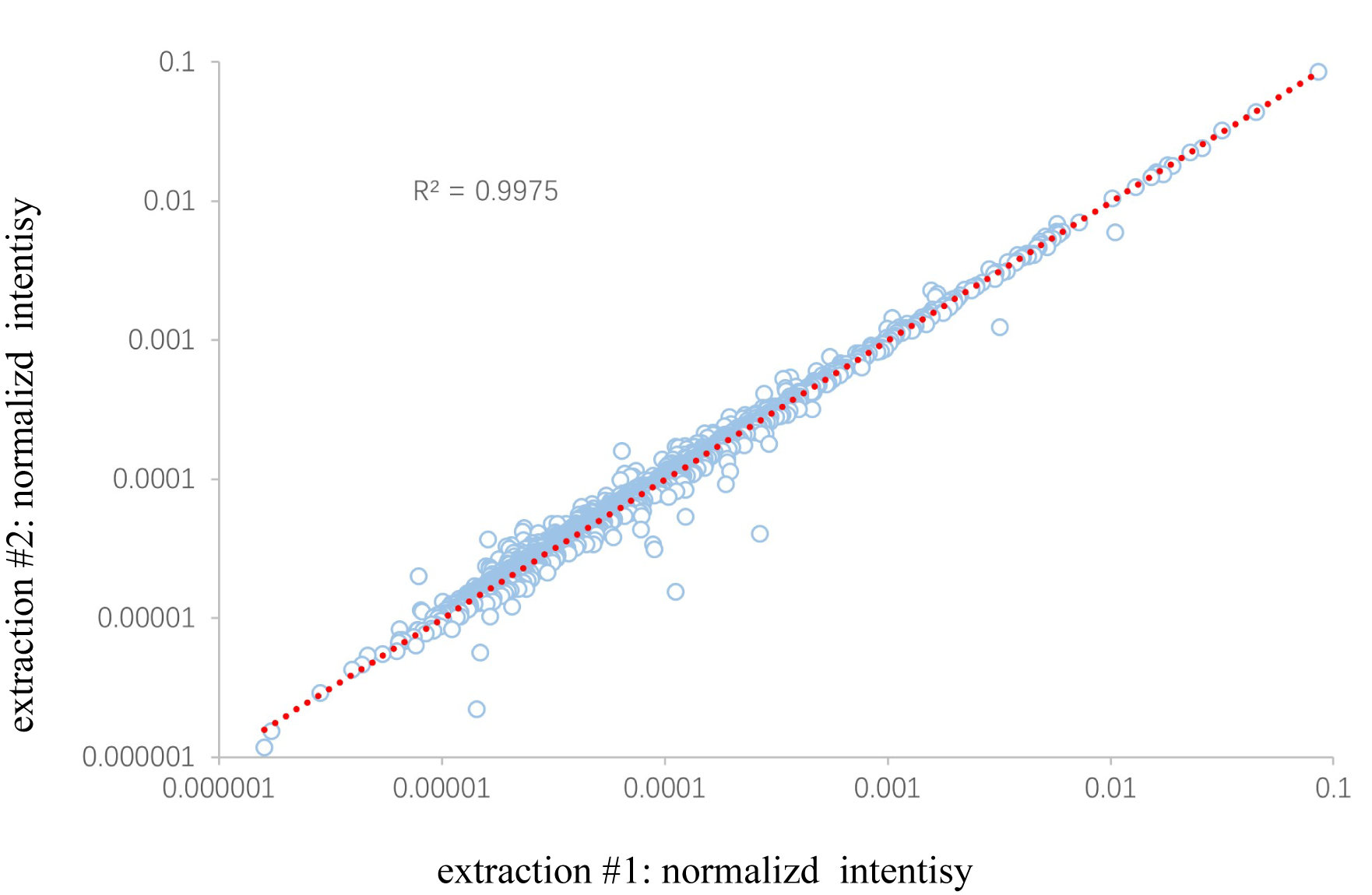


Figure S3 Partial least squares discriminant analysis (PLS–DA) of tea samples. A, The PLS-DA score plot. B, Permutation plot for PLS-DA model.


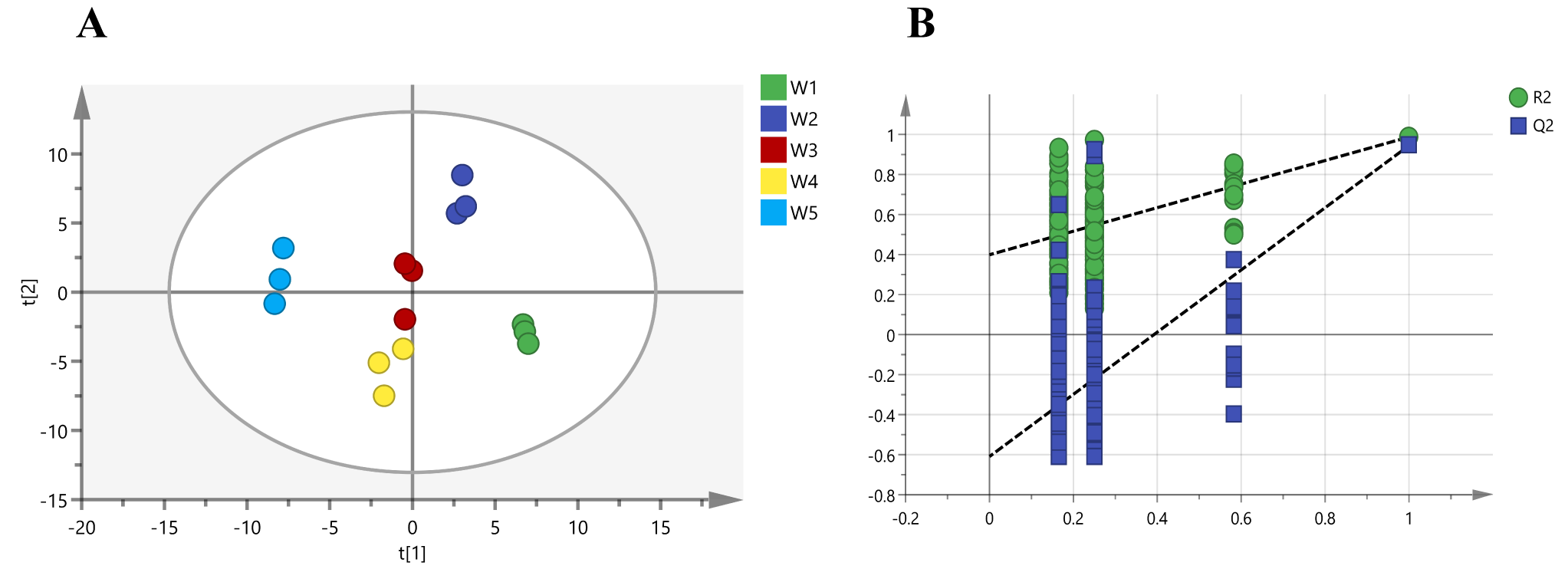


Figure S4 The total amount of each metabolite class. Different letters indicate a significant difference evaluated by one-way ANOVA with LSD post hoc test. *p* < 0.05 is considered as significant. Data was presented using normalized intensity (×10^5^).


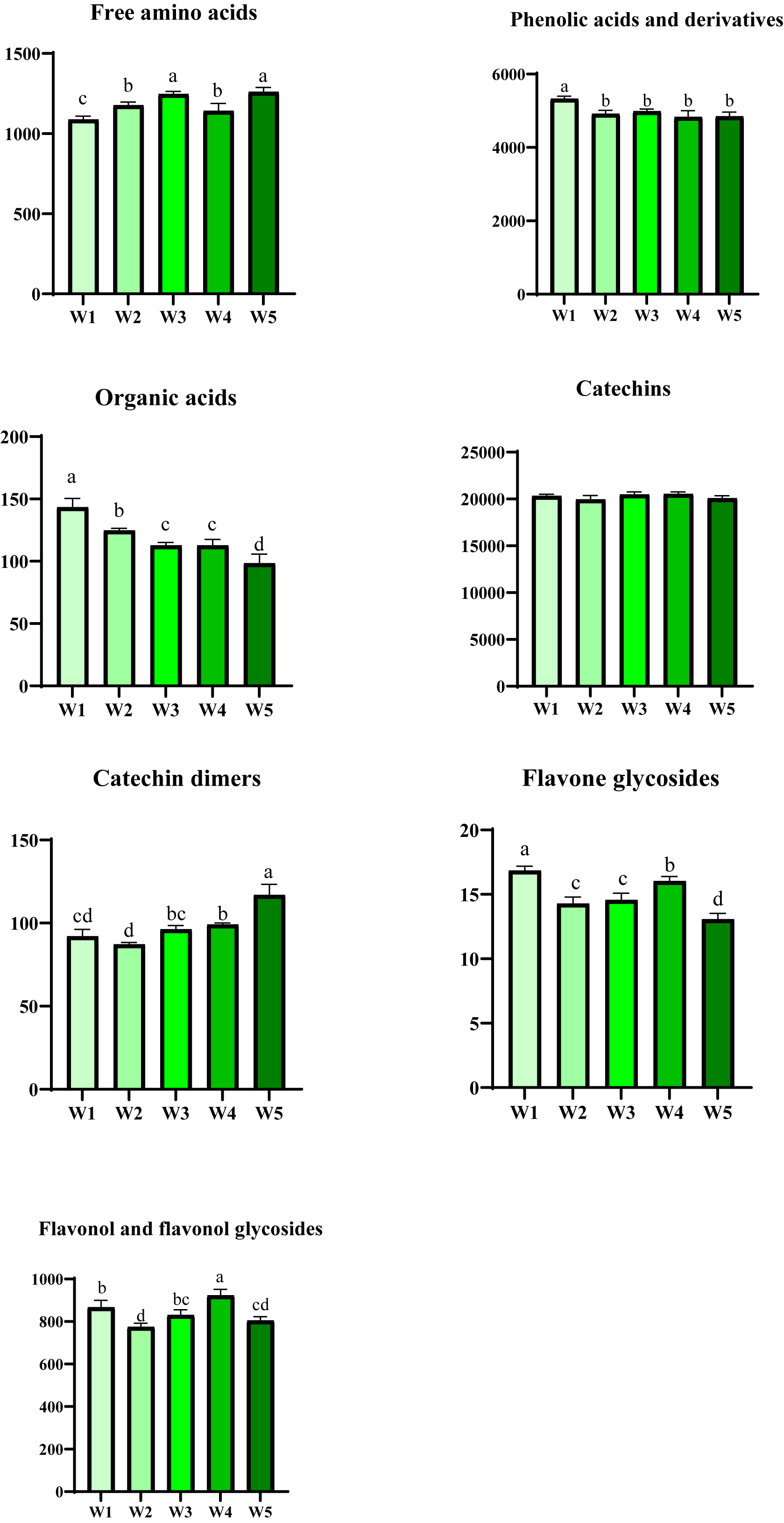

Supplement: Supplementary file 1 [file Data_Sheet_1.docx]
